# Multimorbidity frameworks impact the composition of patterns and their associations with patient-reported outcomes among people with HIV

**DOI:** 10.1177/26335565251331732

**Published:** 2025-04-04

**Authors:** Luxsena Sukumaran, Alan Winston, Jane Anderson, Marta Boffito, Frank A. Post, Memory Sachikonye, Patrick W. G. Mallon, Laura Waters, Jaime Vera, Fiona Burns, Caroline A. Sabin

**Affiliations:** 1 Institute for Global Health, 204274 University College London, London, UK; 2 National Institute for Health and Care Research (NIHR) Health Protection Research Unit (HPRU) in Blood-borne and Sexually Transmitted Infections at University College London, London, UK; 3 Department of Infectious Disease, 156433 Imperial College London, London, UK; 4 4721 Homerton University Hospital, London, UK; 5 9762 Chelsea and Westminster Healthcare NHS Foundation Trust, London, UK; 6 8948 King’s College Hospital NHS Foundation Trust, London, UK; 7 UK Community Advisory Board (UK-CAB), London, UK; 8 8797 University College Dublin, Dublin, Ireland; 9 Mortimer Market Centre, Central and North West London NHS Foundation Trust, London, UK; 10 12190 Brighton and Sussex Medical School, Brighton, UK

**Keywords:** multimorbidity, comorbidity, HIV, multimorbidity patterns

## Abstract

**Objectives:** There is no consensus definition for multimorbidity. We explored how different frameworks affect multimorbidity patterns and their associations with patient-reported outcomes using the prospective, observational Pharmacokinetic and clinical Observations in PeoPle over fiftY (POPPY) Study.

**Methods:** Sixty-four conditions were classified into three frameworks: Framework-D (diseases), Framework-DCI (diseases and clinical indicators) and Framework-DCIS (diseases, clinical indicators and symptoms). Principal component analysis (PCA) identified five comparable patterns: *Cardiovascular disease (CVD)*, *Sexually transmitted diseases*, *Metabolic/AIDS-related*, *Mental health/Other*, and *Cancer*. A sixth pattern was identified using Framework-D (*Infections/Skin)* and Framework-DCI/DCIS (*Cardiometabolic)*. Using PCA loadings, burden z-scores were calculated for each individual/pattern, and their associations with functional impairment (Lawton Instrumental Activities of Daily Living <8), hospitalisation and SF-36 physical and mental health scores were assessed using logistic or linear regression.

**Results:** The analyses included 1073 people with HIV (median [interquartile range; IQR] age 52 [47 - 59] years; 85% male; 97% on ART). Clinical indicators and symptoms were correlated with the *CVD*, *Cardiometabolic* and *Mental health/Other* patterns. While differences were marginal, Framework-DCI showed slightly stronger relationships between *CVD* and functional impairment, hospitalisation and physical health compared to Framework-D. Similarly, Framework-DCIS demonstrated somewhat stronger associations between *Metabolic/AIDS-related* and *Mental health/Other* patterns with certain outcomes.

**Conclusions:** The inclusion of clinical indicators and symptoms were associated with some changes in the strength of associations between certain multimorbidity patterns and outcomes. Our findings suggest that their inclusion in multimorbidity frameworks should be guided by the specific research context and question, rather than solely by effect size on patient-important outcomes.

## Introduction

In the current antiretroviral treatment (ART) era, multimorbidity has become an important HIV research priority due to its rising prevalence and associations with adverse health outcomes.^1–4^ In the context of HIV, multimorbidity can be defined as the presence of one or more health conditions that co-occur alongside an individual’s HIV infection. The disproportionately higher burden of multimorbidity in people with HIV has been shown to greatly reduce quality of life, and increase functional disability, healthcare utilization/costs and mortality.^5–8^ These negative outcomes are further exacerbated by current clinical guidelines and treatments, which often target individual diseases independently. This absence of integrated and coordinated care can lead to higher healthcare costs, polypharmacy and adverse drug interactions.^9,10^

The identification of the most common patterns of multimorbidity, i.e. groups of conditions that are more likely to co-occur within the same individual (beyond that which would be expected by chance), can lead to the generation of new hypotheses on underlying aetiology and inform the development of patient-oriented guidelines for the prevention and management of multimorbidity,^11^ for instance, by identifying specific groups of individuals who would benefit from targeted interventions. However, our recent scoping review of studies among people with HIV highlighted the lack of consensus in the measurement of multimorbidity patterns, with individual studies varying in their design (e.g. the type and number of conditions included) and statistical approach.^11^ Moreover, the extent to which the composition of data-driven patterns differs based on the conditions considered remains unclear. Different multimorbidity frameworks have been proposed that can guide studies that use simple or weighted counts of the morbidities that are present, but their application to studies that use data-driven approaches has not yet been explored.^12–14^ Using data from the Canadian Longitudinal Study on Aging (CLSA), Griffith and colleagues explored how the category of conditions (i.e. diseases, risk factors and symptoms) included in multimorbidity frameworks affected prevalence estimates (using a simple count approach) and patient-important outcomes.^15^ The authors found that frameworks that included risk factors increased prevalence estimates, while those that included symptoms increased prevalence estimates and was linked to greater associations with patient-important outcomes. This study, along with others, suggested that risk factors may be highly prevalent but may have little impact on an individual’s current health outcomes (e.g. quality of life and functional status). In contrast, symptoms may be more strongly associated with current illness burden and health outcomes. Although multiple reviews have highlighted the challenges of their inclusion,^14,16,17^ the addition of risk factors and symptoms to measures of multimorbidity may provide a more holistic understanding of its importance in a given population.

The present study aimed to compare data-driven patterns identified using three different multimorbidity frameworks, as previously described by Griffith and colleagues,^15^ in a cohort of people with HIV receiving clinical care in the UK and Ireland. Specific objectives were to examine whether the choice of framework affects the composition of multimorbidity patterns and their associations with patient-important outcomes, and to consider the implications of each framework for clinical practice.

## Methods

### Study design/population

The Pharmacokinetic and clinical Observations in PeoPle over fiftY (POPPY) study is a prospective observational study that aims to examine the effects of ageing on the clinical outcomes of people with HIV in the UK and Ireland. The characteristics and eligibility criteria of the POPPY cohort have been described previously.^18^ Briefly, two cohorts of people living with HIV were recruited from seven outpatient clinics in England and one in Ireland between April 2013 and January 2016 (baseline visit): an ‘older’ group aged 50 years or older (n=699) and a ‘younger’ group aged 18-49 years (n=374; frequency-matched to the older group on sex, ethnicity, sexual orientation and clinic). The study also recruited a group of people without HIV aged 50 years or older (n=304) who were not included in the present analysis. All participants provided written informed consent. The study was approved by the UK National Research Ethics Service (NRES; Fulham London, UK number 12/LO/1409).

### Data on comorbidities

This study uses data on comorbidities (defined here as coexisting conditions among participants with HIV) collected at baseline. As previously described,^18^ information on the presence or absence of comorbidities was collected using self-reported medical history, that was obtained during structured interviews conducted by trained research staff and, where possible, confirmed using concomitant medication (nonantiretroviral) and healthcare utilisation data (including visits to general practitioners, hospitals, psychiatrists/psychologists, specialists, and use of ambulance/hospital transport). Participants were asked to report whether they had ever developed any of the 52 health conditions listed in the questionnaire, and to add any other conditions experienced (using free-text questions). In order to obtain clinically interpretable patterns of morbidities, we considered 70 comorbidities (from 19 organ system/pathogenic groups) that had a prevalence ≥1.5% in the study population.

### Multimorbidity frameworks

We adapted the frameworks proposed by Griffith and colleagues,^15^ labelling ‘risk factors’ as ‘clinical indicators’ to align with our study’s scope, as we did not consider traditional risk factors such as smoking and alcohol consumption. Thus, the three frameworks employed included: diseases only (Framework-D), diseases and clinical indicators (Framework-DCI), and diseases, clinical indicators, and symptoms (Framework-DCIS). Similar to Griffith et al, we used the proposed categorisation in Willadsen et al’s review to categorise each condition in the three frameworks as a ‘disease’, ‘clinical indicator’ or ‘symptom’.^14^ Diseases were defined as an entity that is associated with a pathological process and is based on either symptoms and/or objective measures (e.g. diabetes or depression). Clinical indicators were defined as conditions or measurements that predict the development of morbidity or mortality, and are not necessarily recognised by the individual. Symptoms were defined as a functional effect that was linked to one or more disease processes (e.g. joint pain is linked to osteoporosis and osteoarthritis). Six of the initial 70 conditions were not categorised into any of the three frameworks after discussions between co-authors and were excluded from the present analyses. The conditions included in each multimorbidity framework are listed in Table 1.

**Table 1.** List of comorbidities considered by category (disease, clinical indicator or symptom) with their prevalence in all POPPY participants with HIV (*n* = 1,073).

Organ system/pathogenic group	Comorbidity	Prevalence (n=1073)	Diseases	Clinical indicator	Symptom	Excluded
AIDS events	Tuberculosis	78 (7.3)	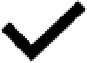			
	Cytomegalovirus	28 (2.6)	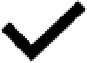			
	Pneumocystis pneumonia	96 (8.9)	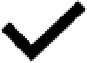			
	Kaposi’s sarcoma	70 (6.5)	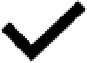			
	AIDS-related cancers	19 (1.8)	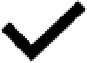			
	Other AIDS events	113 (10.5)				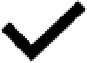
Infections	Varicella zoster virus	90 (8.4)	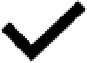			
	Fungal infection	52 (4.8)	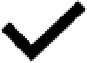			
Endocrine	Type 2 diabetes	56 (5.2)	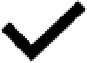			
	Lipodystrophy/lipoatrophy	27 (2.5)	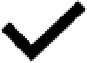			
	Dyslipidaemia	304 (28.3)	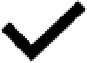			
	Hypothyroidism	42 (3.9)	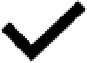			
	Hypogonadism/low testosterone	26 (2.4)	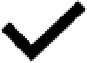			
Mental health	Clinical depression	346 (32.2)	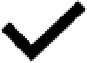			
	Depressive symptoms	84 (7.8)			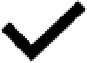	
	Anxiety/panic attacks	96 (8.9)	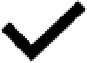			
	Sleeping problems	76 (7.1)			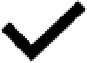	
	Psychosis	18 (1.7)			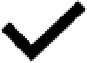	
Nervous system	Dizziness/vertigo	116 (10.8)			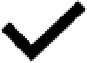	
	Loss of consciousness	31 (2.9)			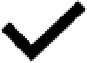	
	Encephalitis	17 (1.6)	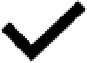			
	Epilepsy	43 (4)	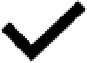			
	Peripheral neuropathy	238 (22.2)	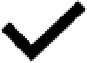			
	Migraine/headaches	52 (4.8)			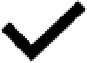	
Haematological	Anaemia	25 (2.3)	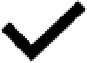			
	Deep vein thrombosis	22 (2.1)	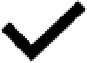			
Cancer	Haematological cancer	13 (1.2)	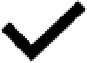			
	Solid organ cancer	55 (5.1)	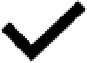			
	Skin cancer	45 (4.2)	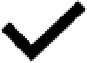			
Cardiovascular	Myocardial infarction	37 (3.4)	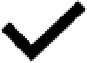			
	Peripheral vascular disease	20 (1.9)	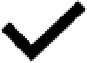			
	Hypertension	245 (22.8)		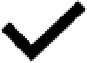		
	Transient ischemic attack	37 (3.4)	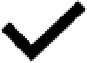			
	Coronary artery bypass grafting	24 (2.2)				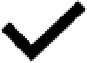
	Heart failure	23 (2.1)	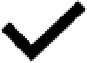			
	Ischaemic heart disease	46 (4.3)	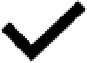			
	Arrhythmia	38 (3.5)	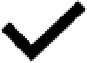			
Hepatitis	Hepatitis A	45 (4.2)	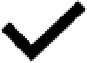			
	Hepatitis B	199 (18.5)	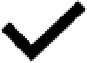			
	Hepatitis C	101 (9.4)	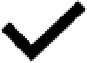			
Gastrointestinal	Persistent bowel disorder	233 (21.7)	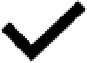			
	Hernia	42 (3.9)				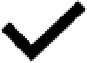
	Gastroesophageal reflux disease	82 (7.6)	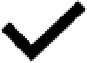			
	Liver diseases	76 (7.1)	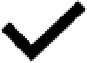			
Genitourinary	Renal problems	54 (5)	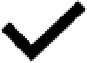			
	Urinary incontinence	43 (4)			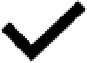	
	Erectile dysfunction	78 (7.3)			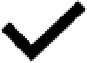	
	Prostate dysfunction	37 (3.4)	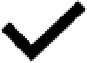			
	Kidney stones	31 (2.9)	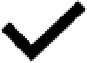			
	Urinary tract infection	34 (3.2)	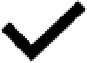			
Bone and joint	Joint inflammation/arthritis	192 (17.9)	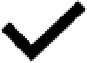			
	Joint replacement	25 (2.3)				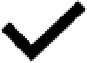
	Osteopenia/osteoporosis	85 (7.9)		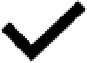		
	Joint/back pain	131 (12.2)			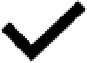	
Sexually transmitted	Syphilis	327 (30.5)	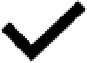			
	Gonorrhoea	458 (42.7)	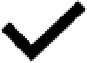			
	Chlamydia	296 (27.6)	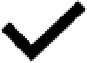			
	Lymphogranuloma venereum	45 (4.2)	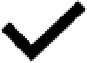			
	Human papilloma virus	105 (9.8)	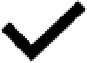			
	Herpes simplex virus	155 (14.4)	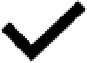			
Respiratory	Asthma/bronchitis/chronic obstructive pulmonary disease, bronchiectasis	263 (24.5)	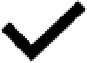			
	Pneumonia	53 (4.9)	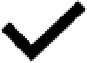			
	Chest infection	115 (10.7)	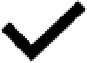			
	Hay fever/allergy	85 (7.9)				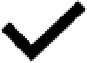
Skin	Psoriasis	49 (4.6)	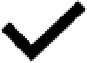			
	Eczema/dermatitis	115 (10.7)	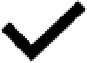			
	Pruritus	21 (2.0)			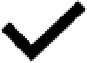	
Ear dysfunction	Ear dysfunction	61 (5.7)	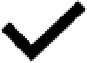			
Eye problem	Eye problem	88 (8.2)	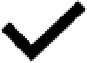			
Vitamin deficiency	Vitamin D deficiency	24 (2.2)				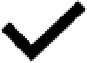
Total			51	3	10	6

### Patient-reported outcomes

Four health-related outcomes were considered: patient-reported physical and mental health, functional impairment and hospitalisation. The Short Form Health Survey (SF-36) questionnaire was used to obtain physical and mental health summary scores.^19^ Briefly, scores from eight subscales were standardised into z-scores (with a mean of 0 and a standard deviation of 1) using sex-specific and age-specific means and standard deviations from the 1996 Health Survey for England dataset,^20^ then combined to obtain a physical and mental health summary score, rescaled with a mean of 50 and a standard deviation of 10. Functional impairment was defined as an impairment in 1 or more activity of daily living using the Lawton Instrumental Activities of Daily Living (IADL) scale.^21^ Hospitalisation (yes or no) in the year preceding the baseline study visit was obtained from information collected on healthcare resource use.

### Statistical analysis

Multimorbidity patterns were identified using Principal Component Analysis (PCA). PCA is a data reduction method that aims to reduce the dimensionality of a dataset, in which there are a large number of interrelated variables, whilst retaining the maximum variance among the original variables. The steps of PCA include: (1) factorisation of the correlation or covariance matrix that measures the pairwise associations between the original variables; (2) extraction of a smaller set of principal components (PCs), which are linear combinations of the original variables and retain the maximum amount of variation in the original dataset; (3) rotation of the PCs to improve interpretability, and (4) selection of the optimal number of PCs that should be retained. These PCs can be interpreted as multimorbidity patterns, i.e. non-random groups of conditions that are frequently associated with each other. Pairwise associations between conditions were assessed using the Somers’ D statistic for binary variables, as proposed by Ng et al.^22^ This pairwise concordance statistic adjusts for expected coincidental conditions by chance and offers higher power to detect non-random associations compared to other measures, including Kendall’s Tau-b, kappa, gamma and adjusted Rand index.^22^ In addition, an *oblimin* rotation was employed to enable patterns to be associated with each other, and therefore allowing multiple patterns to be present within the same individual. The optimal (most interpretable) number of PCs were determined using scree plot and the very simple structure (VSS) criterion.^23^

Multimorbidity patterns were identified using the three frameworks (Framework-D, Framework-DCI, and Framework-DCIS). We then compared the PCs (or patterns) identified using the different frameworks and the conditions with a correlation ≥0.25 with each pattern (also used to determine the label of the pattern). Burden scores were calculated for each participant/pattern using data on the conditions (presence/absence) that also had a correlation ≥0.25 with the corresponding PC/pattern. This threshold was chosen as conditions that met this threshold were found to be interconnected and clinically associated with one another (based on discussions with clinical members of the study team). We assessed overall (box plot illustrating distribution of raw scores, with median (interquartile range, IQR) reported) and individual-level differences (changes in an individuals’ rank were visualised using a Sankey plot) in burden scores across the three frameworks.

The associations of each burden z-score (standardised relative to pattern/framework mean) with physical/mental summary scores (n=881) were assessed using linear regression and regression coefficients: β coefficients represent the increase/decrease in the physical/mental score associated with a one-standard deviation (SD) increase in the burden score of a pattern. Associations of the scores with functional impairment (Lawton IADL <8; n=1020) and hospitalisation (n=1073) were assessed using logistic regression and odds ratios (ORs) are reported. All models adjusted for age and gender. A significance level of p<.05 guided statistical interpretation.

Sensitivity analyses were conducted to determine whether there were any considerable differences in multimorbidity patterns when using a stricter PCA threshold of ≥0.40. The composition of the patterns was compared with those based on a PCA correlation threshold ≥0.25.

The analyses were performed in all POPPY participants with HIV using R V4.2.4 (R Foundation for Statistical Computing, Vienna, Austria).

## Results

### Characteristics of study participants

The sociodemographic, lifestyle and HIV-related characteristics of POPPY participants are summarised in Table 2. POPPY participants were predominantly male (85.2%), of white ethnicity (84.1%), men who have sex with men (MSM; 76.1%) with a median (IQR) age of 52 (47, 59) years. The majority of participants were on ART (97.5%) and had an undetectable viral load (<50 copies/mL; 91.3%). The median (IQR) nadir CD4+ T-cell count and years since HIV diagnosis were 202 (100, 304) cells/μL and 13.2 (7.8, 20.4) years, respectively.

**Table 2.** Baseline socio-demographic, lifestyle and HIV-related characteristics among all POPPY participants with HIV (n=1073).

Characteristic *n (%) or median (IQR)*	All PLWH (n=1073)
Age (years)	52 (47 - 59)
Gender	
Male	914 (85.2)
Female	159 (14.8)
Ethnicity	
Black-African	171 (15.9)
White	902 (84.1)
Sexual orientation	
MSM	816 (76.1)
Heterosexual	257 (24.0)
BMI (kg/m^2^)	25.7 (23.3 - 28.6)
Smoking status	
Never	439 (40.9)
Past	365 (34.0)
Current	269 (25.1)
Alcohol use	
Never	90 (8.4)
Past	125 (11.7)
Current	858 (78.0)
Years since HIV diagnosis	13.2 (7.8 - 20.4)
CD4+ T-cell count (cells/mm^3^)	625 (475 - 811)
Undetectable viral load (<50 copies)	975 (91.3)
Nadir CD4+ count (cells/mm^3^)	202 (100 - 304)
On ART	1046 (97.5)
Years on ART	9.5 (4.9 - 16.3)

### Individual comorbidities

At baseline, 97.9% of all POPPY participants with HIV reported ≥1 comorbidity (median [IQR] per individual: 5 [3, 8]) at baseline, from the original list of 70 comorbidities. When considering only the 52 diseases, 95.6% of all POPPY participants reported ≥1 comorbidity, with a median [IQR] of 4 [2-7]. The prevalence of comorbidities ranged from 1.0% (pancreatitis) to 42.7% (gonorrhoea) (Table 1). Other prevalent comorbidities included clinical depression (32.2%), syphilis (30.5%) and dyslipidaemia (28.3%).

### Composition of multimorbidity patterns

The three frameworks, Framework-D, Framework-DCI, and Framework-DCIS, incorporated 51, 54 and 64 conditions, respectively. Six patterns were identified across the three frameworks, explaining 27.2% (Framework-D), 27.7% (Framework-DCI) and 24.8% (Framework-DCIS) of the total variation. Of which, five were comparable across the frameworks: *Cardiovascular disease (CVD)*, *Sexually transmitted diseases (STDs)*, *Metabolic/AIDS-related*, *Mental health/Other*, and *Cancer* (Table 3). A sixth pattern was also identified: *Infections/Skin* (Framework-D) and *Cardiometabolic* (Framework-DCI and Framework-DCIS). In all three frameworks, the *CVD* pattern (variability ranged from 2.9-4.0%) showed strong correlations between ischemic heart disease, heart failure, peripheral vascular disease, myocardial infarction and renal problems, with the addition of hypertension (Framework-DCI and Framework-DCIS), anaemia (Framework-DCI) and epilepsy (Framework-DCIS). Across all three frameworks, the same conditions were identified in the *STDs* (gonorrhoea, chlamydia, *lymphogranuloma venereum* (LGV), herpes simplex virus (HSV) and syphilis) and *Cancer* (Haematological cancer, Solid organ cancer, AIDS-related cancer) patterns. Using Framework-DCIS, osteopenia/osteoporosis was also highly correlated with the *Cancer* pattern. In the *Mental health*/*Other* and *Metabolic/AIDS-related* patterns, similar conditions were identified using both Framework-D and Framework-DCI. However, strong correlations were observed between specific symptoms (*Mental health*/*Other:* sleeping problems and psychosis; *Metabolic/Infections*: pruritis, dizziness/vertigo, loss of consciousness, urinary incontinence) and these patterns when Framework-DCIS was used. Using Framework-D, an *Infections/Skin* pattern was identified that exhibited strong correlations between conditions including fungal infections, deep vein thrombosis, psoriasis, peripheral vascular disease, HPV and hepatitis A. This pattern was not observed when using Framework-DCI or DCIS. Instead, a *Cardiometabolic* pattern (dyslipidaemia, hypertension, lipodystrophy/lipoatrophy and type II diabetes) was identified. These conditions were also strongly correlated with myocardial infarction when Framework-DCI was used.

**Table 3.** Patterns identified using PCA in all POPPY participants with HIV (n=1,073) using three frameworks: (1) Framework-D (diseases only, n=52 comorbidities), (2) Framework-DCI (diseases and clinical indicators, n=54 comorbidities), and (3) Framework-DCIS (diseases, clinical indicators and symptoms, n=64 comorbidities).

PC (% of variance explained)	Label	Comorbidities with correlation ≥0.25 (correlation with PC)
Framework-D – Diseases only (27.2% of total variation)		
1 (6.9%)	Metabolic/AIDS-related	Peripheral neuropathy (0.65), Hypothyroidism (0.48), CMV (0.44), type II diabetes (0.43), PCP (0.38), KS (0.38), Eye problems (0.36), Skin cancer (0.33), Asthma/ Bronchitis/COPD (0.29), CVA/TIA (0.28), HSV (0.28)
2 (5.5%)	STDs	Gonorrhoea (0.79), syphilis (0.66), Chlamydia (0.66), LGV (0.62), HSV (0.50) hepatitis C (0.37), hepatitis A (0.37)
3 (4.2%)	Cancer	Haematological cancer (0.97), Solid organ cancer (0.91), AIDS-related cancers (0.39)
4 (4.0%)	CVD	IHD (0.70), heart failure (0.68), peripheral vascular disease (0.54), myocardial infarction (0.50), renal problems (0.42)
5 (3.5%)	Mental health/Other	Clinical depression (0.76), Anxiety/Panic attacks (0.49), joint inflammation/Arthritis (0.39), Persistent bowel disorders (0.37), Asthma/Bronchitis/COPD (0.36), Chest infections (0.36), Lipodystrophy/Lipoatrophy (0.30), anaemia (0.29), Eczema/Dermatitis (0.27), Hypogonadism (0.26), Encephalitis (0.26)
6 (3.2%)	Infections/Skin	Fungal infections (0.33), DVT (0.43), Anxiety/Panic attacks (0.34), psoriasis (0.34), PVD (0.33), HPV (0.29), hepatitis A (0.28)
Framework-DCI – Diseases and clinical indicators (27.7% of total variation)		
1 (7.3%)	Cardiometabolic	**Dyslipidaemia** (0.74), **hypertension** (0.57), Lipodystrophy/Lipoatrophy (0.52), type II diabetes (0.48), myocardial infarction (0.44)
2 (5.7%)	STDs	Gonorrhoea (0.78), syphilis (0.68), Chlamydia (0.65), LGV (0.62), HSV (0.51), hepatitis C (0.37), hepatitis A (0.36)
3 (4.2%)	Cancer	Haematological cancer (0.96), Solid organ cancer (0.89), AIDS-related cancers (0.40)
4 (3.9%)	Mental health/Other	Clinical depression (0.74), Anxiety/Panic attacks (0.50), Persistent bowel disorders (0.40), joint inflammation/Arthritis (0.37), Asthma/ Bronchitis/COPD (0.35), Chest infections (0.35), anaemia (0.33), Encephalitis (0.32), Hypogonadism (0.26)
5 (3.4%)	Metabolic/AIDS-related	Peripheral neuropathy (0.57), CMV (0.58), PCP (0.45), Eye problems (0.45), Hypothyroidism (0.42), KS (0.30), TB (0.30), type II diabetes (0.27), Pneumonia (0.26)
6 (3.2%)	CVD	IHD (0.65), heart failure (0.64), peripheral vascular disease (0.61), **hypertension** (0.47), myocardial infarction (0.40), renal problems (0.42), anaemia (0.28)
Framework-DCIS – Diseases, clinical indicators and symptoms (24.8% of total variation)		
1 (6.5%)	Cardiometabolic	Dyslipidaemia (0.66), Lipodystrophy/Lipoatrophy (0.44), type II diabetes (0.37), hypertension (0.34)
2 (4.8%)	STDs	Gonorrhoea (0.79), syphilis (0.66), Chlamydia (0.64), LGV (0.64), HSV (0.49), hepatitis C (0.37), hepatitis A (0.35)
3 (3.9%)	Metabolic/AIDS-related	Peripheral neuropathy (0.62), CMV (0.47), PCP (0.42), pruritis (0.47), Hypothyroidism (0.46), type II diabetes (0.39), Eye problems (0.39), dizziness/vertigo (0.38), KS (0.26), Pneumonia (0.26), loss of consciousness (0.26), urinary incontinence (0.26), Ear dysfunction (0.26)
4 (3.6%)	Cancer	Haematological cancer (0.97), Solid organ cancer (0.85), AIDS-related cancers (0.39), Osteopenia/Osteoporosis (0.28)
5 (3.1%)	Mental health/Other	Clinical depression (0.80), Anxiety/Panic attacks (0.52), Asthma/ Bronchitis/COPD (0.37), sleeping problems (0.36), joint inflammation/Arthritis (0.33), Persistent bowel disorders (0.33), Eczema/Dermatitis (0.31), Lipodystrophy/Lipoatrophy (0.31), psychosis (0.30), Chest infections (0.27), Hypogonadism (0.26)
6 (2.9%)	CVD	IHD (0.69), heart failure (0.69), hypertension (0.64), peripheral vascular disease (0.55), myocardial infarction (0.52), renal problems (0.43), epilepsy (0.26)

CVD; Cardiovascular disease, IHD; Ischemic heart disease, STDs; Sexually transmitted diseases, LGV; lymphogranuloma venereum, CMV; Cytomegalovirus, PCP; Pneumocystis pneumonia, MI; myocardial infarction, HSV; herpes simplex virus.

The PCA loadings/coefficients for conditions were used to calculate burden scores for the patterns that were comparable across the three frameworks (Table 3). The distribution of *Metabolic/AIDS-related*, *STDs*, *Mental health/Other* and *Cancer* scores were similar across the three frameworks (Supplemental Figure 1). In contrast, a larger proportion of individuals reported high *CVD* scores when Framework-DCIS (median, interquartile range (IQR): 0 [0, 0.64]) was used compared to Framework-D (0 [0.00, 0.00]). Additionally, the median (IQR) for Cardiometabolic scores was higher for Framework-DCI (0 [0, 0.74]) compared to Framework-DCIS (0 [0, 0.66]). Individual-level differences in burden scores across frameworks were visualised using a Sankey plot (Supplemental Figure 2).

### Associations with patient-reported outcomes

Here, we focused on patterns where we observed strong correlations with clinical indicators and/or symptoms: Framework-DCI (*CVD* and *Cardiometabolic*) and Framework-DCIS (*Metabolic/AIDS-related* and *Mental health/Other*).

**Figure 1.**
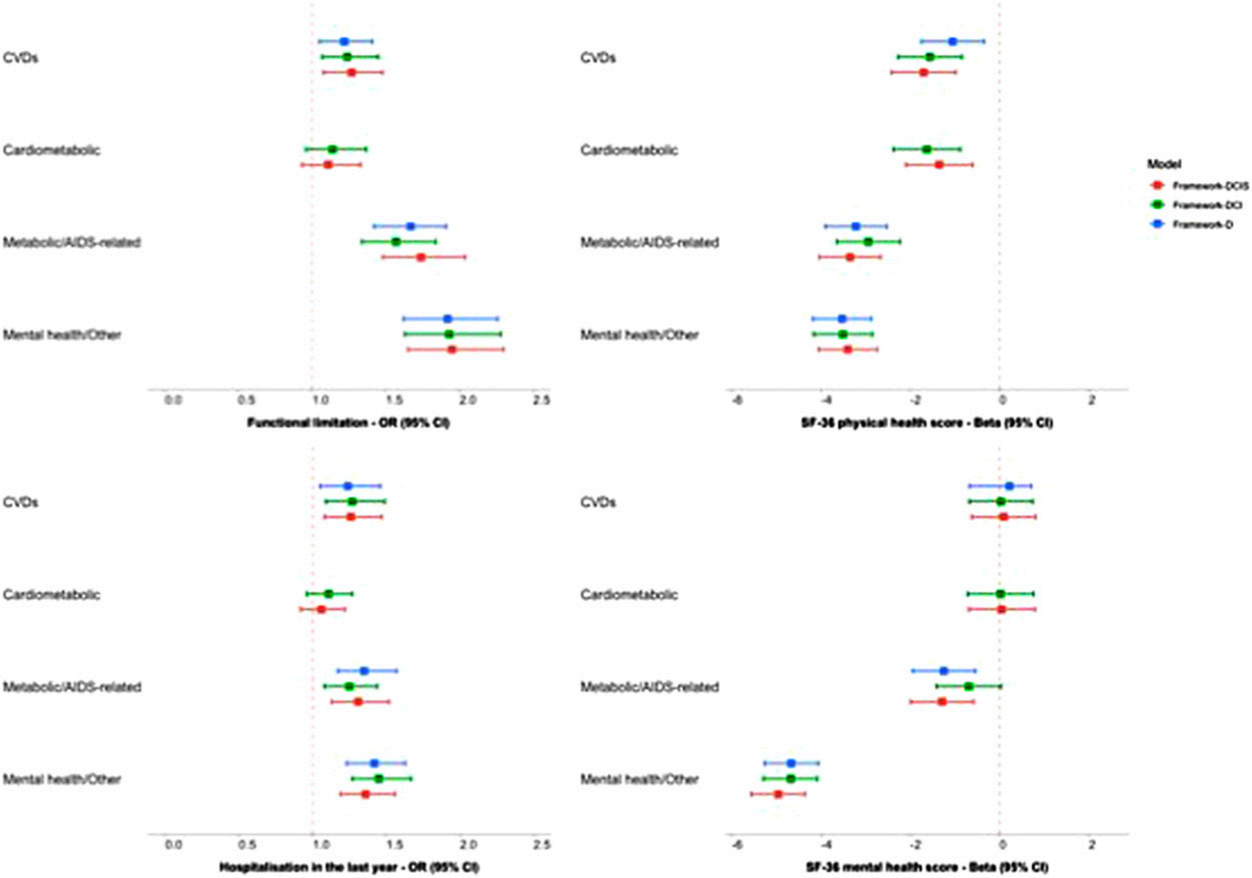
Associations between multimorbidity burden z-scores (based on PCA threshold >0.25) and four outcomes (functional limitation, hospitalisation, and SF-36 physical and mental summary scores), adjusted for age and gender. Odds ratio (OR) or Beta estimates, with 95% confidence intervals (CIs) are presented for the three multimorbidity frameworks (including diseases only [Framework-D], diseases and clinical indicators [Framework-DCI], and diseases, clinical indicators and symptoms [Framework-DCIS]). Sample size for each outcome: functional limitation (n = 1020), hospitalisation (n = 1073), SF-36 physical and mental health (n = 881).

The inclusion of clinical indicators (Framework-DCI) was associated with a slightly stronger relationship between CVD z-scores and functional impairment (1.26 [1.09, 1.46]), compared to the disease-only framework (1.22 [1.04, 1.41]) (Figure 1). Similar trends were observed with this pattern/framework and the odds of hospitalisation in the past year. Using Framework-DCI, CVD z-scores (−1.41 [-2.11, −0.72]) were also associated with significantly poorer physical health scores, compared to Framework-D (−0.94 [-1.64, −0.25]. Although CVD z-scores (using Framework-DCI) was associated with higher mental health, this association did not reach statistical significance (p>0.05). Although the Cardiometabolic z-scores using Framework-DCI could not be directly compared with Framework-D (not identified using this framework), we found that higher Cardiometabolic z-scores were significantly associated with higher odds of hospitalisation (1.19 [1.04, 1.36], p=0.01) and poorer physical health scores (−1.28 [-1.97, −0.58], p<0.001).

The inclusion of clinical indicators and symptoms (Framework-DCIS) showed a stronger association between Metabolic/AIDS-related z-scores and functional impairment (1.73 [1.49, 2.02]), compared to when Framework-D (1.64 [1.40, 1.91]) or Framework-DCI (1.57 [1.35, 1.83]) were used. In contrast, the association between Mental health/Other z-scores and functional impairment was comparable across the three frameworks. A 1-SD increase in Metabolic/AIDS-related z-scores (based on Framework-DCIS) was associated with 36% higher odds of hospitalisation (1.36 [1.18, 1.57]), compared to 30% when Framework-DCI (1.30 [1.13, 1.49]) was used, respectively. In contrast, a 1-SD increase in Mental health/Other z-score (using Framework-DCIS) was associated with 38% higher odds of hospitalisation, compared to 43% and 47% when both Framework-D and Framework-DCI were used, respectively (all p<0.001). A similar trend was also observed between Mental Health/Other z-scores and the SF-36 physical summary scores. However, using Framework-DCIS, Mental health/Other (−4.91 [-5.52, −4.31]) z-scores were significantly associated with poorer mental health scores, compared to both Framework-D (−4.61 [-5.22, −3.99]) or Framework-DCI (Mental health/Other: −4.63 [-5.24, −4.02]).

### Sensitivity analysis

A smaller number of conditions was associated with the patterns when a PCA threshold of ≥0.40 was used (Supplemental Table 1). Using this cut-off, hypertension (clinical indicator) remained strongly correlated with the *CVD* pattern. However, only one symptom (pruritis) was observed to be correlated with the *Metabolic/AIDS-related* pattern. Other symptoms that were previously observed with this pattern using a PCA threshold ≥0.25, as well as the *Mental health/Other* pattern, did not meet the PCA threshold ≥0.40. Given the small number of conditions and the lack of clinical indicators/symptoms associated with patterns using a PCA threshold >0.40, we decided not to examine burden scores using this threshold and their impact on the four health outcomes.

## Discussion

The present study explored the impact of different frameworks on multimorbidity patterns and their associations with patient-reported outcomes in a cohort of people with HIV receiving clinical care in the UK and Ireland.

We found that the choice of framework affects the composition of the multimorbidity patterns identified. Although five similar patterns were identified across all three frameworks, the inclusion of clinical indicators and symptoms revealed an additional *Cardiometabolic* pattern. Both clinical indicators and symptoms were also found to be correlated with the following patterns: *CVD*, *Metabolic/AIDS-related* and *Mental health/Other*, reflecting the broad spectrum of co-occurring morbidity that is experienced by this population. This is also supported by previous studies that have investigated multimorbidity patterns among people living with HIV, which have found strong correlations between clinical indicators (e.g. hypertension) and/or symptoms (e.g. sleeping problems and dizziness/vertigo) and specific patterns such as CVD and mental health/neurological problems.^24–27^

The associations between multimorbidity burden scores and patient-reported outcomes also differed depending on the framework used. We observed that by including clinical indicators and symptoms, some diseases were no longer strongly correlated (correlation ≥0.25) with the respective pattern. We also found that the inclusion of clinical indicators and symptoms generally resulted in stronger associations compared to Framework-D, but this effect was not consistent across all outcomes. The inclusion of clinical indicators was associated with stronger relationships between *CVD* and *Metabolic/AIDS-related* patterns with functional impairment, hospitalisation and physical health compared to when only diseases were considered, highlighting their potential relevance in understanding patient health beyond traditional disease-centric models. However, the associations between *Mental Health*/Other burden z-scores and hospitalisation, as well as physical health, were stronger using Framework-D than Framework-DCIS, suggesting that the specific symptoms that were strongly correlated (sleeping problems and psychosis) may have little impact on these outcomes. These findings suggest that the inclusion of clinical indicators and symptoms may not strengthen associations in all contexts, and thus the decision to include these factors in multimorbidity frameworks should be predominately driven by the research context and study objectives, rather than solely by associations with outcomes.

While these findings indicate that effect size estimates differ across frameworks, it is important to interpret these variations in a conceptually meaningful way. The aim of comparing frameworks is not simply to establish whether differences in effect sizes reach statistical significance, but rather to assess whether these differences can impact study conclusions. Given that different frameworks assign varying weights to conditions with distinct etiological and clinical profiles, some degree of variation in effect sizes is expected. However, the observed trends, such as the strengthening of associations when symptoms and clinical indicators are included, suggest that some of these differences may not be random. Instead, they likely reflect the multidimensional nature of multimorbidity and the complex interplay between diseases, clinical indicators, and symptoms. This aligns with findings from general population studies, where including symptoms such as pain and depressive symptoms has been more strongly associated with poorer self-rated health than diseases themselves among individuals living with multimorbidity.^28^ Additionally, Griffith et al found that including symptoms in the definition of multimorbidity (defined as two or more chronic conditions) led to stronger associations with disability, and self-rated physical and mental health, compared to a disease-only framework.^15^ However, these studies use a simple count approach to measure multimorbidity and therefore do not take into account conditions that are more likely to co-occur (non-random) due to shared aetiologies and interactions.

We also found that the three frameworks had little/no impact on the composition of the *STDs* and *Cancer* patterns. This suggests that not all multimorbidity patterns are equally affected by the inclusion of clinical indicators and symptoms. Further work is needed to understand whether including a wider range of clinical indicators and symptoms, that may be more relevant to these patterns, exhibit stronger correlations and subsequently affect their associations with health outcomes.

Our findings revealed two important methodological considerations for future research. Firstly, PCA loading thresholds influence which conditions are included in each pattern. We found that often symptoms were correlated with patterns at a lower PCA loading (around 0.25) compared to diseases. Therefore, we selected a threshold of ≥0.25, and by doing so, we highlighted the importance of including symptoms through the associations between burden scores, generated using Framework-DCIS, and health outcomes. However, a higher threshold of ≥0.40 would have excluded symptoms and the true associations between certain patterns and health outcomes may have not been reflected. Secondly, PCA thresholds are arbitrary, and the inclusion of clinical indicators and symptoms can lead to subtle or substantial shifts in the loadings/coefficients of any of the conditions that were previously associated with a pattern (examples can be seen in Supplemental Table 2-8). This, however, does not necessarily signify that the condition is no longer associated with the pattern, but rather underscores the need for researchers to visualise and interpret how the coefficients of the conditions change across different frameworks. By doing so, researchers can then decide on whether conditions should be included based on the magnitude of change and clinical relevance. This should ultimately be guided by the specific research focus/scope of the study, as well as the outcomes of interest. Different frameworks can serve different purposes. For instance, Framework-DCI may be more useful than Framework-D, particularly in preventive contexts. For example, in preventive cardiology, this framework can reveal how factors such as hypertension cluster with cardiovascular disease, allowing clinicians to identify high-risk groups and focus on targeted prevention. In contrast, Framework-DCIS provides a more holistic view of health by incorporating symptom burden, which is particularly valuable in settings where symptom management significantly impacts quality of life and care needs.

To our knowledge, this study represents one of the first efforts to describe how the category of conditions affects the composition of multimorbidity patterns and their associations with health outcomes. However, our study has some limitations. First, data on comorbidities were collected using self-reported questionnaires which may introduce recall bias, and lead to under-reporting and/or misclassification of certain comorbidities. This may be particularly true for symptoms that were predominately collected through free-text questions. However, where possible, information on comorbidities was supported using healthcare utilisation and concomitant medication data. Second, there is subjectivity in categorising diseases, clinical indicators and symptoms. However, to be consistent with previous publications, we adopted the categorisation used in Willadsen et al’s systematic review, and consulted with clinicians to categorise any conditions that were not included in their review. Third, we included comorbidities with a prevalence ≥1.5% in the cohort to ensure we could capture the diverse/wide spectrum of comorbidities typically reported in this population, as well as reduce the impact of random noise when identifying patterns. However, expanding the spectrum of diseases, clinical indicators and symptoms included may have improved our understanding of how the categories of conditions affect patterns and patient-important outcomes. Fourth, the associations between burden scores and health outcomes are presented after adjusting for age and gender, as an initial step in understanding these relationships. Future analyses should explore other potential confounders, such as socioeconomic status and lifestyle factors. while adjusting for relevant confounders e.g. age and gender. Fifth, there were other important outcomes that were beyond the scope of this manuscript (e.g. polypharmacy, healthcare costs, frailty and death) and may have potentially been affected by the different multimorbidity frameworks. Therefore, further work in this area is needed.

In summary, this study underscores the importance of considering a broad spectrum of conditions, including clinical indicators and symptoms, to gain a more comprehensive and holistic understanding of health outcomes beyond traditional disease-centric frameworks. We emphasise the importance of interpreting differences in effect sizes through a clinical and conceptual lens, recognising that framework selection should be guided by the specific research question and the outcomes of interest. By doing so, clinicians and researchers can gain valuable insight into the complex interplay between different comorbidities and their impact on patient outcomes, ultimately guiding more tailored strategies to manage multimorbidity and improve patient care for those living with HIV.

## Supplemental Material

Supplemental Material - Multimorbidity frameworks impact the composition of patterns and their associations with patient-reported outcomes among people with HIV Supplemental Material for Multimorbidity frameworks impact the composition of patterns and their associations with patient-reported outcomes among people with HIV by Luxsena Sukumaran, Alan Winston, Jane Anderson, Marta Boffito, Frank A Post, Memory Sachikonye, Patrick WG Mallon, Laura Waters, Jaime Vera, Fiona Burns, and Caroline A Sabin in Journal of Multimorbidity and Comorbidity

## Data Availability

The authors confirm that the data supporting the findings of this study are available within the article and/or its supplementary materials. *
