# Supplementary material for: Multimorbidity frameworks impact the composition of patterns and their associations with patient-reported outcomes among people with HIV
Source: J Multimorb Comorb. 2025 Apr 4;15:26335565251331732. doi: 10.1177/26335565251331732 (PMC11970071; doi:10.1177/26335565251331732)
Supplement: Supplemental Material - Multimorbidity frameworks impact the composition of patterns and their associations with patient-reported outcomes among people with HIV [file sj-pdf-1-cob-10.1177_26335565251331732.pdf]

SUPPLEMENTARY MATERIAL

Supplementary Table 1. All comorbidities with a correlation ≥0.40 with corresponding PCs or patterns

PC (% of variance explained)	Label	Comorbidities with correlation ≥0.25 (correlation with PC)
Framework-D – Diseases only (27.2% of total variation)		
1 (6.9%)	Metabolic/AIDS-related	Peripheral neuropathy (0.65), Hypothyroidism (0.48), CMV (0.44), Type II diabetes (0.43)
2 (5.5%)	STDs	Gonorrhoea (0.79), Syphilis (0.66), Chlamydia (0.66), LGV (0.62), HSV (0.50)
3 (4.2%)	Cancer	Haematological cancer (0.97), Solid organ cancer (0.91)
4 (4.0%)	CVDs	IHD (0.70), Heart failure (0.68), Peripheral vascular disease (0.54), Myocardial infarction (0.50), Renal problems (0.42)
5 (3.5%)	Mental health/Other	Clinical depression (0.76), Anxiety/Panic attacks (0.49)
6 (3.2%)	Infections/Skin	DVT (0.43)
Framework-DCI – Diseases and clinical indicators (27.7% of total variation)		
1 (7.3%)	Cardiometabolic	Dyslipidemia (0.74), Hypertension (0.57), Lipodystrophy/Lipoatrophy (0.52), Type II diabetes (0.48), Myocardial infarction (0.44)
2 (5.7%)	STDs	Gonorrhoea (0.78), Syphilis (0.68), Chlamydia (0.65), LGV (0.62), HSV (0.51)
3 (4.2%)	Cancer	Haematological cancer (0.96), Solid organ cancer (0.89), AIDS-related cancers (0.40)
4 (3.9%)	Mental health/Other	Clinical depression (0.74), Anxiety/Panic attacks (0.50), Persistent bowel disorders (0.40)
5 (3.4%)	Metabolic/AIDS-related	Peripheral neuropathy (0.57), CMV (0.58), PCP (0.45), Eye problems (0.45), Hypothyroidism (0.42)
6 (3.2%)	CVDs	IHD (0.65), Heart failure (0.64), Peripheral vascular disease (0.61), Hypertension (0.47), Renal problems (0.42), Myocardial infarction (0.40)
Framework-DCIS – Diseases, clinical indicators and symptoms (24.8% of total variation)		
1 (6.5%)	Cardiometabolic	Dyslipidemia (0.66), Lipodystrophy/Lipoatrophy (0.44)
2 (4.8%)	STDs	Gonorrhoea (0.79), Syphilis (0.66), Chlamydia (0.64), LGV (0.64), HSV (0.49)
3 (3.9%)	Metabolic/AIDS-related	Peripheral neuropathy (0.62), CMV (0.47), PCP (0.42), Pruritis (0.47), Hypothyroidism (0.46)
4 (3.6%)	Cancer	Haematological cancer (0.97), Solid organ cancer (0.85)
5 (3.1%)	Mental health/Other	Clinical depression (0.80), Anxiety/Panic attacks (0.52)
6 (2.9%)	CVDs	IHD (0.69), Heart failure (0.69), Hypertension (0.64), Peripheral vascular disease (0.55), Myocardial infarction (0.52), Renal problems (0.43)

CVDs; Cardiovascular diseases, IHD; Ischemic heart disease, STDs; Sexually transmitted diseases, LGV; Lymphogranuloma venereum, CMV; Cytomegalovirus, PCP; Pneumocystis pneumonia, MI; Myocardial infarction, HSV; Herpes simplex virus

Supplementary Figure 1. Distribution of patterns' burden scores across the three frameworks using a PCA threshold of ≥0.25: (1) Framework-D (diseases only; green), (2) Framework-DCI (diseases and clinical indicators; blue), and (3) Framework-DCIS (diseases, clinical indicators and symptoms; orange)

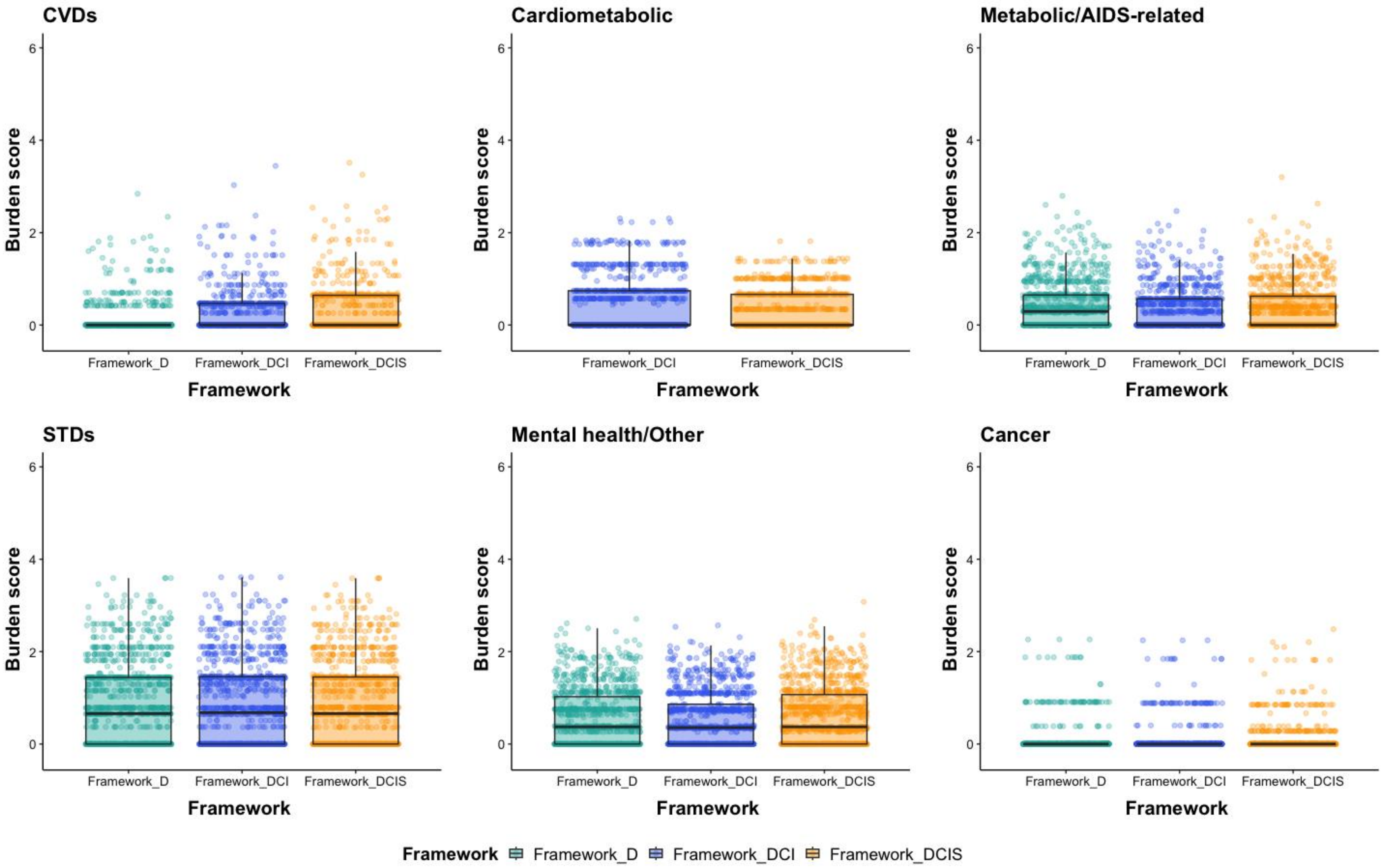

Supplementary Figure 2. Individuals were ranked based on their burden scores and grouped into quintiles (1- lowest and 5 - highest) for each pattern/framework. Individual-level changes in ranks are highlighted in this Sankey plot.

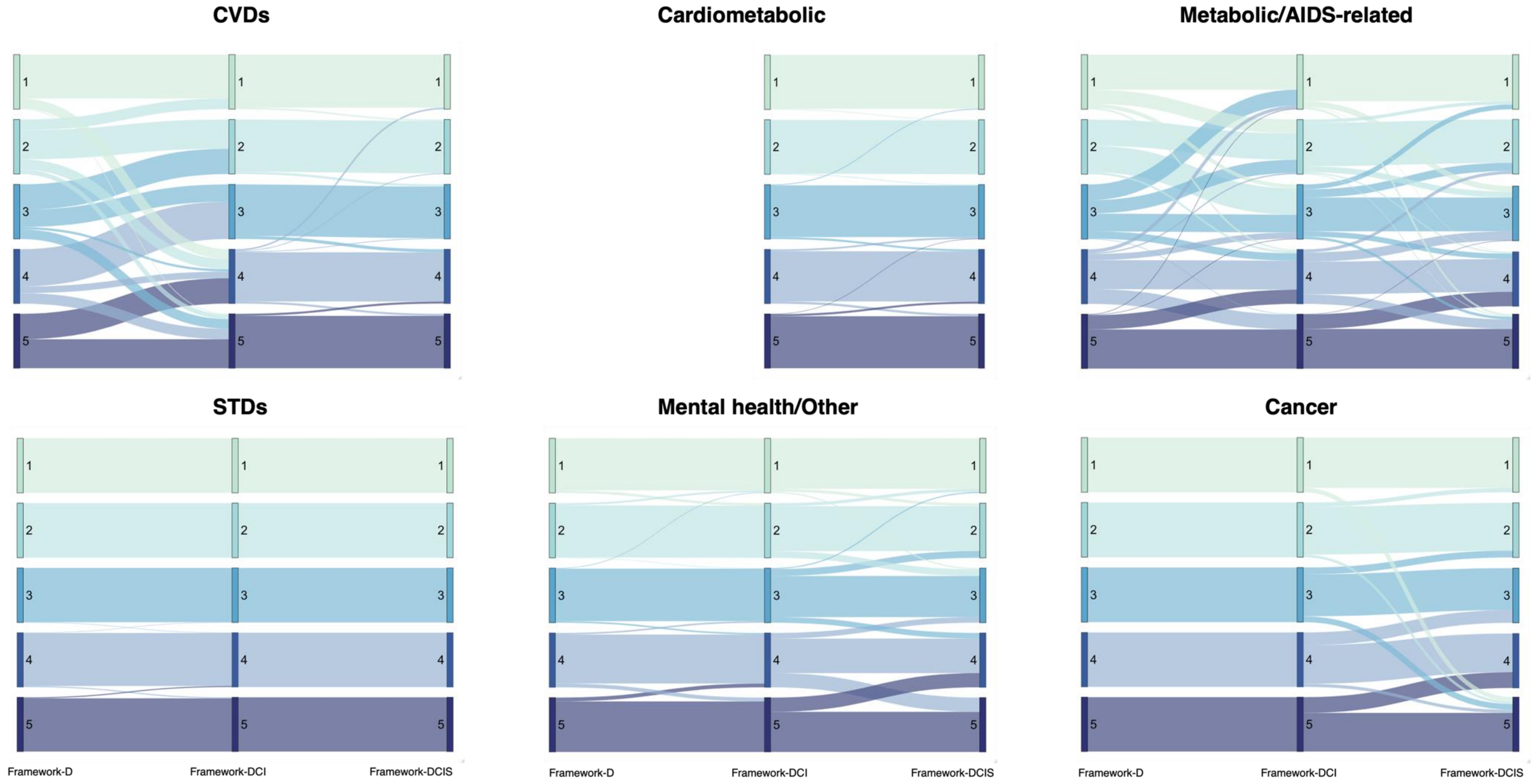

Supplementary Table 2. All comorbidities with a correlation ≥0.20 with the *CVDs* pattern; correlation: 0.20-0.24 (orange); 0.25-0.29 (teal); 0.30-0.39 (blue); ≥0.40 (dark blue)

Comorbidities	Framework-D	Framework-DCI	Framework-DCIS
Dyslipidemia			0.21
Anaemia	0.24	0.28	0.21
Epilepsy	0.20	0.26	0.26
Peripheral neuropathy	0.21		
MI	0.50	0.40	0.52
IHD	0.70	0.65	0.69
Peripheral vascular disease	0.54	0.61	0.55
Heart failure	0.68	0.64	0.69
Hypertension		0.47	0.64
CVA/TIA	0.24		0.25
Pneumonia		0.23	0.24
Liver diseases			
Renal problem	0.42	0.40	0.43

Supplementary Table 3. All comorbidities with a correlation ≥0.20 with the *Cardiometabolic* pattern; correlation: 0.20-0.24 (orange); 0.25-0.29 (teal); 0.30-0.39 (blue); ≥0.40 (dark blue)

Comorbidities	Framework-DCI	Framework-DCIS
Kaposi's sarcoma		0.21
VZV	0.23	
Type II diabetes	0.48	0.37
Dyslipidemia	0.74	0.66
Lipodystrophy/lipoatrophy	0.52	0.44
Peripheral neuropathy	0.24	
Myocardial infarction	0.44	0.21
Hypertension	0.57	0.34
Erectile dysfunction		0.22
Psoriasis	0.24	
Eczema/Dermatitis	0.21	
Skin cancer	0.22	0.24

Supplementary Table 4. All comorbidities with a correlation >0.25 with the *Metabolic/ AIDS-related* pattern; correlation: 0.20-0.24 (orange); 0.25-0.29 (teal); 0.30-0.39 (blue); ≥0.40 (dark blue)

Comorbidities	Framework-D	Framework-DCI	Framework-DCIS
TB	0.25	0.30	0.21
CMV	0.44	0.58	0.47
Pneumocystis pneumonia	0.38	0.45	0.42
Kaposi's sarcoma	0.38	0.30	0.26
Type II diabetes	0.43	0.27	0.39
Hypogonadism	0.21		
Hypothyroidism	0.48	0.42	0.46
Dizziness/Vertigo			0.38
Loss of consciousness			0.26
Encephalitis		0.23	0.23
Peripheral neuropathy	0.65	0.57	0.62
CVA/TIA	0.26	0.22	0.23
Asthma/Bronchitis/COPD/bronchiectasis	0.29		0.24
Pneumonia	0.22	0.26	0.21
Hepatitis B		0.21	0.24
Kidney stones	0.23		
Urinary Incontinence			0.26
Prostate dysfunction			0.22
Joint inflammation/Arthritis	0.23		
Osteopenia/Osteoporosis		0.24	
HSV	0.28	0.22	0.21
Pruritis			0.47
Eye problem	0.36	0.45	0.39
Ear dysfunction	0.21	0.21	0.26
Skin cancer	0.33	0.22	

Supplementary Table 5. All comorbidities with a correlation >0.25 with the *STDs* pattern; correlation: 0.20-0.24 (orange); 0.25-0.29 (teal); 0.30-0.39 (blue); $\geq$0.40 (dark blue)

Comorbidities	Framework-D	Framework-DCI	Framework-DCIS
Arrhythmia		0.20	
Hepatitis B		0.20	
Hepatitis C	0.37	0.37	0.37
Hepatitis A	0.37	0.36	0.35
Urinary Incontinence			0.21
Syphilis	0.66	0.68	0.66
Gonorrhea	0.79	0.78	0.79
Chlamydia	0.66	0.65	0.64
LGV	0.62	0.62	0.64
HSV	0.50	0.51	0.49

Supplementary Table 6. All comorbidities with a correlation >0.25 with the *Mental health/Other* pattern; correlation: 0.20-0.24 (orange); 0.25-0.29 (teal); 0.30-0.39 (blue); $\geq$0.40 (dark blue)

Comorbidities	Framework-D	Framework-DCI	Framework-DCIS
Hypogonadism	0.26	0.26	0.26
Lipodystrophy/Lipoatrophy	0.30	0.23	0.31
Anaemia	0.29	0.33	0.23
Clinical depression	0.76	0.74	0.80
Anxiety/Panic attacks	0.49	0.50	0.52
Sleeping problems			0.36
Psychosis			0.30
Dizziness/Vertigo			0.23
Encephalitis	0.26	0.32	0.22
Peripheral vascular disease		0.21	
Asthma/Bronchitis/COPD/bronchiectasis	0.36	0.35	0.37
Chest infections	0.36	0.36	0.27
Persistent bowel disorders	0.37	0.40	0.33
GORD	0.24	0.22	0.23
Hepatitis C	0.21	0.20	0.21
Liver diseases	0.22	0.24	
Joint inflammation/Arthritis	0.39	0.37	0.33
Eczema/Dermatitis	0.27	0.24	0.31

Supplementary Table 7. All comorbidities with a correlation >0.25 with the *Cancer* pattern; correlation: 0.20-0.24 (orange); 0.25-0.29 (teal); 0.30-0.39 (blue); $\geq$0.40 (dark blue)

Comorbidities	Framework-D	Framework-DCI	Framework-DCIS
AIDS-related cancers	0.39	0.40	0.39
Depressive symptoms			0.21
Osteopenia/Osteoporosis		0.24	0.28
Haematological cancer	0.97	0.96	0.97
Solid organ cancer	0.91	0.89	0.85

Supplementary Table 8. All comorbidities with a correlation >0.25 with the *Infections/Skin* pattern; correlation: 0.20-0.24 (orange); 0.25-0.29 (teal); 0.30-0.39 (blue); $\geq$0.40 (dark blue)

Comorbidities	Framework-D
CMV	0.22
Fungal infection	0.33
Deep vein thrombosis	0.43
Anxiety/Panic attacks	0.34
Peripheral vascular disease	0.33
Hepatitis A	0.28
Renal problem	0.24
Kidney stones	0.25
HSV	0.29
Psoriasis	0.24
Eczema/Dermatitis	0.22
